# Importância Prognóstica do Eletrocardiograma Pré-Operatório em Pacientes de Baixo Risco Submetidos à Intervenção Cirúrgica sob Anestesia Geral

**DOI:** 10.36660/abc.20230098

**Published:** 2024-01-24

**Authors:** Lafayete Ramos, Alexandre Chataubriand Coutinho, José Rebelato, Marcos Vinicius Ramos, Eliane Elly, Pedro Amoedo, Gustavo Viel, Valdir Ambrósio Moises

**Affiliations:** 1 Instituto Brasileiro de Controle do Câncer São Paulo SP Brasil Instituto Brasileiro de Controle do Câncer , São Paulo , SP – Brasil; 2 Universidade Federal de São Paulo São Paulo SP Brasil Universidade Federal de São Paulo , São Paulo , SP – Brasil; 3 Centro Universitário São Camilo São Paulo SP Brasil Centro Universitário São Camilo , São Paulo , SP – Brasil; 4 Faculdade de Medicina de Marilia Marília SP Brasil Faculdade de Medicina de Marilia , Marília , SP – Brasil

**Keywords:** Eletrocardiografia, Cuidados Pré-Operatórios, Cirurgia Geral

## Abstract

**Fundamento:**

Pacientes com idade superior a 50 anos requerem quatro vezes mais intervenções cirúrgicas que o grupo mais jovem. Muitas diretrizes recomendam a realização do eletrocardiograma pré-operatório nessa faixa etária.

**Objetivos:**

Determinar a importância do ECG pré-operatório em pacientes com idade superior a 50 anos e com classificação de risco cirúrgico ASA I e II.

**Métodos:**

Foram recrutados pacientes com idade superior a 50 anos, sem comorbidades, submetidos à intervenção cirúrgica sob anestesia geral. Os pacientes foram randomizados para a realização (grupo A n=214) ou não (grupo B n=213) do ECG pré-operatório. Foram analisadas as variáveis: sexo, idade, resultado do ECG, da radiografia do tórax e dos exames laboratoriais, risco cirúrgico, duração do procedimento, eventos adversos e mortalidade intra-hospitalar. O nível de significância estatística adotado foi de 5%.

**Resultados:**

Houve ocorrência de desfechos adversos em 23 (5,4%) pacientes, com um número significante de eventos adversos nos pacientes do sexo masculino (OR=7,91, IC95% 3,3-18,90, p<0,001) e naqueles com intervenções de maior porte cirúrgico (OR=30,02, IC95% 4,01-224,92, p<0,001). Não houve diferença entre os grupos que realizaram ou não o ECG (OR=1,59, IC95% 0,67-3,75, p=0,289). As demais variáveis não mostraram diferenças significantes. Na regressão logística multivariada o sexo masculino (OR=6,49; IC95% 2,42-17,42, p<0,001) e o porte cirúrgico (OR=22,62; IC95% 2,95-173,41, p=0,002) foram preditores independentes de desfechos adversos, enquanto realizar ou não ECG (OR=1,09; IC95% 0,41-2,90, p=0,867) permaneceu sem significância estatística.

**Conclusões:**

Os resultados sugerem que o ECG pré-operatório não foi capaz de predizer aumento do risco de desfechos adversos nos pacientes estudados, durante a fase hospitalar.

## Introdução

O número de procedimentos cirúrgicos não cardíacos está em franca ascensão, ultrapassando a barreira das 300 milhões de intervenções ao ano. ^1 , 2^ Nos países desenvolvidos, a mortalidade hospitalar varia de 0,4 a 0,8% e a taxa de complicações entre 3% e 16%, das quais 40% estão relacionadas ao sistema cardiovascular. ^3 , 4^ Além disso, a população acima dos 50 anos de idade requer quatro vezes mais intervenções cirúrgicas que o grupo mais jovem. Ainda, com o envelhecimento crescente da população, graças principalmente a uma melhor atenção dada às enfermidades crônicas, é estimado que exista um aumento exponencial dos procedimentos nesse grupo nos próximos anos. ^5^

Atualmente recomenda-se a realização de um eletrocardiograma (ECG) pré-operatório em indivíduos com idade superior a 40 anos, visto que estudos na população cirúrgica mostram que alterações eletrocardiográficas aumentam com a idade e muitas delas podem ter implicações clínicas para o manuseio anestésico. ^6 , 7^

Devido a isso, na maioria das instituições, faz-se rotineiramente avaliação clínica e laboratorial antes de uma cirurgia para determinar a condição pré-operatória do paciente com o objetivo de reduzir a morbimortalidade. E no âmbito da avaliação clínica, um dos exames realizados é o ECG. Esse é particularmente solicitado em pacientes acima dos 40 anos de idade independente da condição clínica, com grau de recomendação variável dependendo da diretriz adotada, mas sempre com um nível de evidência fraco, exatamente pela escassez de estudos com número de pacientes, desenho e qualidade suficientes para uma recomendação mais robusta. ^8 - 12^

O presente estudo visa avaliar pela primeira vez, em um desenho prospectivo e randomizado, a necessidade da realização de um ECG na rotina pré-operatória para pacientes com idade superior a 50 anos, sem comorbidades, e que foram submetidos à intervenção cirúrgica não cardíaca de forma eletiva sob anestesia geral.

## Métodos

Entre abril 2020 e fevereiro 2022 foram avaliados 427 pacientes com idade superior a 50 anos sem comorbidades, que foram submetidos à intervenção cirúrgica sob anestesia geral. Todos possuíam exame físico normal no momento da avaliação clínica pré-operatória. Os pacientes que preencheram os critérios de inclusão e aceitaram participar do estudo após assinar o termo de consentimento livre e esclarecido foram randomizados na proporção 1:1 para a realização ou não do ECG.

Todos os pacientes realizaram exames laboratoriais (hemograma, glicemia, ureia, creatinina e coagulograma) e radiografia de tórax na posição posteroanterior, e foram submetidos a procedimento cirúrgico sob anestesia geral para tratamento de doença neoplásica.

Foram coletadas de cada paciente as seguintes variáveis: sexo, idade, resultado do ECG (para aqueles que realizaram), da radiografia do tórax e dos exames laboratoriais (hemograma, glicemia, ureia, creatinina e coagulograma). Informações sobre o risco cirúrgico pré-operatório (American Society of Anesthesiology - ASA), duração do procedimento, eventos adversos e mortalidade intra-hospitalar também foram contabilizados. A morbimortalidade intra-hospitalar dos pacientes que fizeram o ECG (grupo A) foi comparada com aqueles que foram submetidos à intervenção cirúrgica sem o exame (grupo B). Os pacientes do grupo A ainda foram divididos em dois subgrupos: A1 (com ECG normal) e A2 (com ECG apresentando alguma anormalidade) que foram comparados entre si e com os pacientes do grupo B. O ECG foi interpretado pelo cardiologista sênior da instituição participante e foi considerado anormal qualquer traçado que apresentasse um ritmo diferente do sinusal, presença de sobrecargas de câmaras cardíacas, bloqueios intraventriculares ou atrioventriculares, inversão da polaridade da onda T em pelo menos duas derivações contíguas, mais que três batimentos prematuros atriais ou ventriculares, presença de pré-excitação ventricular ou QTc > 470 ms. Radiografia de tórax foi avaliada por dois radiologistas do departamento de imagem e foi considerada anormal a presença de infiltrados parenquimatosos, sequela de tuberculose, presença de derrame pleural, área cardíaca aumentada ou presença de metástases. Foi considerado exame laboratorial alterado qualquer resultado diferente dos limites considerados normais adotados pelo laboratório central da Instituição. Nenhum paciente foi excluído da randomização devido a alguma anormalidade observada nos exames laboratoriais, na radiografia de tórax ou no ECG.

O porte cirúrgico foi dividido em dois grandes grupos: baixo e moderado/alto risco. Para efeito desse estudo foi considerada cirurgia de moderado/alto risco qualquer procedimento intracavitário (crânio, tórax, abdome ou pelve) ou aqueles cuja reposição de fluidos durante o ato cirúrgico excederam 30 mL/Kg.

Desfecho adverso foi considerado qualquer tipo de complicação (clínica e/ou cirúrgica) que aumentasse o tempo de internação previsto para cada procedimento realizado ou óbito por qualquer causa os quais foram estudados de forma individual e em conjunto sob denominação de morbimortalidade.

Esse estudo foi aprovado pelo Comitê de Ética e Pesquisa do Instituto Brasileiro de Controle do Câncer e pelos comitês das instituições coparticipantes sob o n ^o^ CAAE 20728719.3.0000.0072

### Análise estatística

As características qualitativas foram descritas em frequências absolutas e relativas segundo a realização ou não do ECG, e verificada a associação pelo teste do qui-quadrado ou testes exatos (teste exato de Fisher ou teste da razão de verossimilhanças). As características quantitativas foram descritas segundo realização do ECG com uso de medidas resumo (média, desvio padrão, mediana e quartis) e comparadas pelo teste t-Student não pareado ou teste Mann-Whitney, conforme normalidade de distribuição avaliada por meio do teste de Kolmogorov-Smirnov.

A ocorrência de morbimortalidade foi descrita segundo cada característica qualitativa e quantitativa pelos mesmos testes mencionados anteriormente.

As variáveis que foram significativas nas análises pelos testes exatos de Fisher/razão de verossimilhança ou teste t-Student/Mann-Whitney relativamente à morbimortalidade foram inseridas na análise multivariada com uso de regressão logística, inserindo o uso de ECG na análise para avaliar se a realização do exame teve influência na morbimortalidade.

As análises foram realizadas com uso do software SPSS for Windows versão 22.0 e os testes foram realizados com nível de significância de 5%.

## Resultados

Esse estudo incluiu 427 pacientes (83.6% mulheres) acima de 50 anos (58,04±6,89 anos -) portadores de tumores sólidos sem história de comorbidades e que foram submetidos a procedimentos cirúrgicos eletivos sob anestesia geral. O grupo que realizou ECG (grupo A) era mais velho, possuía mais anormalidades nos exames laboratoriais e foi submetido a procedimento com maior tempo operatório. As demais variáveis estudadas não apresentaram diferenças significativas ( Tabela 1 ).

**Tabela 1:** – Análise da população que foi submetida à intervenção cirúrgica com e sem eletrocardiograma (ECG)

Variável	ECG						
	
	Não realizado		Realizado		Total		p
	
	n	%	n	%	n	%	
**Sexo**							0,199
Masculino	30	14,1	40	18,7	70	16,4	
Feminino	183	85,9	174	81,3	357	83,6	
**Idade (anos)**	57,37±6,16		58,71±7,51		427	100,0	0,044 ^#^
**Porte Cirúrgico**							0,225
Baixo Risco	123	57,7	110	51,9	233	54,8	
Moderado/Alto Risco	90	42,3	102	48,1	192	45,2	
**RX Tórax**							0,192
Normal	208	97,7	204	95,3	412	96,5	
Anormal	5	2,3	10	4,7	15	3,5	
**Laboratório**							<0,001
Normal	213	100,0	199	93,0	412	96,5	
Anormal	0	0,0	15	7,0	15	3,5	
**Risco Cirúrgico (ASA)**							0,372*
I	212	99,5	210	98,1	422	98,8	
II	1	0,5	4	1,9	5	1,2	
**Tempo Cirúrgico(h)**	2 (2 – 3)		2 (2 – 4)		427	100,0	0,042 ^$^
**Mortalidade**							>0,999*
Não	212	99,5	212	99,1	424	99,3	
Sim	1	0,5	2	0,9	3	0,7	
**Complicações revertidas**							0,193
Não	205	96,2	200	93,5	405	94,8	
Sim	8	3,8	14	6,5	22	5,2	
**Morbimortalidade**							0,289
Não	204	95,8	200	93,5	404	94,6	
Sim	9	4,2	14	6,5	23	5,4	
**Total**	213	100	214	100	427	100	

Teste do qui-quadrado; ^*^ Teste exato de Fisher; ^#^ Teste t-Student; ^$^ Teste Mann-Whitney (intervalo interquatil).

Efeitos adversos ocorreram em 23 (5.4%) pacientes, sendo três óbitos (0.7%). Foi observado um número significante de eventos adversos nos pacientes do sexo masculino e naqueles que se submeteram a intervenções de moderado/alto risco, não observando qualquer diferença em relação as complicações pós-operatórias entre os grupos que realizaram ou não o ECG ( Figura Central ). Não foram observadas diferenças estatisticamente significativas em relação à idade, anormalidades observadas no RX de tórax ou laboratoriais, risco cirúrgico (ASA) e duração do procedimento ( Tabela 2 ).

**Figura Central:**
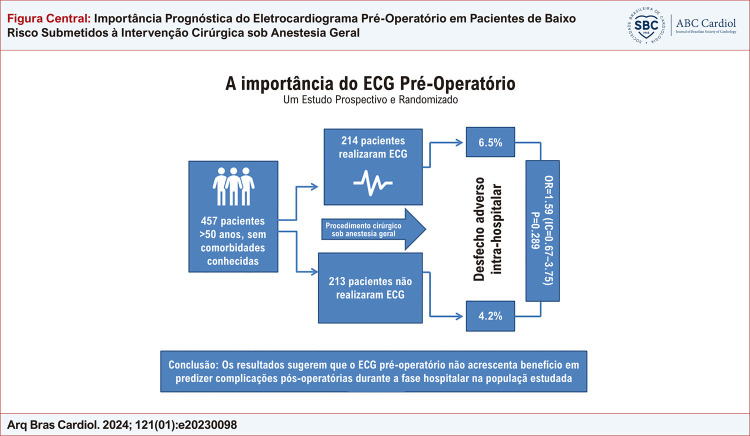
: Importância Prognóstica do Eletrocardiograma Pré-Operatório em Pacientes de Baixo Risco Submetidos à Intervenção Cirúrgica sob Anestesia Geral

**Tabela 2:** – Análise dos desfechos pós-operatórios em relação às variáveis estudadas

Variável	Morbimortalidade							

	Não		Sim		OR	IC (95%)		p
	
	n	%	n	%		Inferior	Superior	
**Sexo**								<0,001*
Masculino	57	81,4	13	18,6	7,91	3,31	18,90	
Feminino	347	97,2	10	2,8	1,00			
**Idade (anos)**	56 (52 – 62)		57 (55 – 60)		1,01	0,95	1,07	0,754
**Porte Cirúrgico**								<0,001
Baixo Risco	232	99,6	1	0,4	1,00			
Moderado/Alto Risco	170	88,5	22	11,5	30,02	4,01	224,92	
**ECG**								0,289
Não realizado	204	95,8	9	4,2	1,00			
Realizado	200	93,5	14	6,5	1,59	0,67	3,75	
**RX Tórax**								>0,999*
Normal	389	94,4	23	5,6	1,00			
Anormal	15	100,0	0	0,0	&			
**Laboratório**								0,190*
Normal	391	94,9	21	5,1	1,00			
Anormal	13	86,7	2	13,3	2,86	0,61	13,52	
**Risco Cirúrgico**								>0,999*
I	399	94,5	23	5,5	1,00			
II	5	100,0	0	0,0	&			
**Tempo Cirúrgico (h)**	2 (2 – 3)		2 (2 – 5)		1,30	1,01	1,67	0,193 ^$^
**Total**	404	94,6	23	5,4				

Teste do qui-quadrado; ^*^ Teste exato de Fisher; ^$^ Teste de Mann-Whitney (intervalo interquartil); IC: intervalo de confiança; & não é possível estimar

Quando se observa a análise de regressão logística multivariada podemos notar que o sexo masculino e o porte cirúrgico foram preditores de desfechos adversos no pós-operatório nessa população, enquanto realizar ou não o ECG permaneceu sem qualquer significância estatística ( Tabela 3 ).

**Tabela 3:** – Preditores de morbimortalidade na população estudada. Análise de regressão logística multivariada

Variável	OR	IC (95%)		p

		Inferior	Superior	
Sexo (masculino)	6,49	2,42	17,42	<0,001
Idade (anos)	1,00	0,93	1,07	0,943
Porte cirúrgico (Moderado/Alto risco)	22,62	2,95	173,41	0,003
Laboratório (Anormal)	1,39	0,22	8,88	0,726
Tempo cirúrgico	1,47	1,09	1,99	0,013
ECG (realizado)	1,09	0,41	2,90	0,867

IC: intervalo de confiança; ECG: eletrocardiograma.

Foi também analisado se a presença de anormalidades eletrocardiográficas impactaria a morbimortalidade pós-operatória. Quando se compara o grupo com anormalidades eletrocardiográficas com aqueles com ECG normal e com o grupo que não foi submetido ao exame (grupo B) não se observa diferença significativa entre os grupos em relação à ocorrência de eventos adversos ( Tabela 4 ). Na comparação entre os pacientes do grupo A, (aqueles em que o ECG era normal) com aqueles que possuíam alguma anormalidade, também não foi observada qualquer diferença ( Tabela 5 ).

**Tabela 4:** – Desfecho pós-operatório em pacientes com eletrocardiograma (ECG) normal ou anormal, comparado com aqueles que não realizaram ECG

Variável	ECG								

	Normal		Anormal		Sem ECG		Total		p

	n	%	n	%	n	%	N	%	
**Mortalidade**									0,241
Não	196	99,5	16	94,1	212	99,5	424	99,3	
Sim	1	0,5	1	5,9	1	0,5	3	0,7	
**Morbidade**									0,300
Não	185	93,9	15	88,2	205	96,2	405	94,8	
Sim	12	6,1	2	11,8	8	3,8	22	5,2	
**Morbimortalidade**									0,402
Não	185	93,9	15	88,2	204	95,8	404	94,6	
Sim	12	6,1	2	11,8	9	4,2	23	5,4	
**Total**	197	100	17	100	213	100	427	100	

Teste da razão de verossimilhança.

**Tabela 5:** – Comparação de desfechos pós-operatórios adversos entre os pacientes com eletrocardiograma (ECG) normal e pacientes com ECG alterado

Variável	ECG				
	
	Normal		Anormal		p
	
	n	%	n	%	
Mortalidade					0,153
Não	196	99,5	16	94,1	
Sim	1	0,5	1	5,9	
**Morbidade**					0,307
Não	185	93,9	15	88,2	
Sim	12	6,1	2	11,8	
**Morbimortalidade**					0,307
Não	185	93,9	15	88,2	
Sim	12	6,1	2	11,8	
**Total**	197	100,0	17	100,0	

Teste da razão de verossimilhança.

Os pacientes do sexo masculino eram mais velhos e foram submetidos à intervenção cirúrgica de maior risco. Em relação as outras variáveis estudadas, não houve diferenças significativas em relação ao gênero ( Tabela 6 ).

**Tabela 6:** – Características dos pacientes em relação ao gênero

Variável	Sexo				

	Masculino		Feminino		p
	
	n	%	n	%	
**ECG**					0,104 ^#^
Normal	34	48,6	163	45,7	
Anormal	6	8,6	11	3,1	
Sem ECG	30	42,9	183	51,3	
**Porte Cirúrgico**					<0,001
Baixo Risco	24	34,8	209	58,7	
Moderado/Alto Risco	45	65,2	147	41,3	
**Tempo Cirúrgico (h)**	2 (1 – 3)		3 (2 – 3)		0,067 ^&^
RX Tórax					
Normal	69	98,6	343	96,1	0,483*
Anormal	1	1,4	14	3,9	
**Laboratório**					0.081*
Normal	65	92,9	347	97,2	
Anormal	5	7,1	10	2,8	
**Risco Cirúrgico (ASA)**					0,593*
I	69	98,6	353	98,9	
II	1	1,4	4	1,1	
**Idade (anos)**	60,9±8,7		57,5±6,3		0,002 ^$^
**Total**	70	100,0	357	100,0	

Teste do qui-quadrado; ^*^ Teste exato de Fisher; ^#^ Teste da razão de verossimilhança; ^$^ teste t-Student; & teste de Mann-Whitney (intervalo interquartil); ASA: American Society of Anesthesiology.

## Discussão

A significância prognóstica do ECG pré-operatório tem sofrido importantes modificações nas últimas décadas. No final dos anos 70 a eletrocardiografia de repouso foi largamente utilizada como marcador de risco cardiovascular em indivíduos submetidos à intervenção cirúrgica eletiva. Alterações eletrocardiográficas como presença de onda Q patológica e vários graus de arritmias faziam parte do escore de risco de Goldman. ^13^ Esses resultados foram posteriormente repetidos, confirmando o valor prognóstico do ECG pré-operatório. ^14 , 15^

Payne et al. ^16^ avaliaram 345 pacientes em uma coorte prospectiva e concluíram que o ECG é um exame útil para predizer eventos cardiovasculares perioperatórios. Outros estudos ^17 - 19^ mostraram que anormalidades no ECG pré-operatório foi capaz de predizer complicações cardiovasculares principalmente quando existe a presença de um QT longo. Entretanto, mais tarde, esses resultados favoráveis foram questionados por vários outros autores, ^6 , 20 , 21^ e essa controvérsia tem se mantido atualmente. Muitas dessas dúvidas poderiam ter sido sanadas se tivéssemos evidências robustas advindas de ensaios prospectivos e randomizados em pacientes submetidos à intervenção cirúrgica eletiva sob anestesia geral.

O principal achado do nosso estudo foi mostrar que pacientes sem comorbidades mesmo acima de 50 anos (idade média de 58 anos) submetidos à intervenção cirúrgica sob anestesia geral parecem não se beneficiar da realização do ECG pré-operatório. Não encontramos diferença no percentual de eventos adversos entre o grupo A, que realizou o ECG quando comparado com o grupo B, sem ECG. Houve até uma tendência de maior morbimortalidade no grupo A, o que pode ser explicado pelo fato de serem mais velhos, possuir mais anormalidade nos exames laboratoriais e de terem realizado procedimentos com maior tempo cirúrgico.

Alguns estudos corroboram nossos achados. Richardson et al., ^21^ avaliando uma coorte de 152 479 pacientes de forma retrospectiva concluiu que o ECG pré-operatório não possui valor para predizer infarto ou mortalidade cardiovascular pós-operatória. Da mesma forma, Liu et al., ^6^ analisando 513 pacientes observaram que anormalidades eletrocardiográficas não era capaz de predizer complicações cardiovasculares na população geriátrica e que esse exame não era útil nesses indivíduos. Na mesma linha, van Klei et al. ^20^ avaliaram o ECG de 2967 pacientes acima de 50 anos em um estudo observacional e notaram que a presença de bloqueios intraventriculares apesar de correlacionar com o risco de infarto pós-operatório e óbito, não melhorou a predição além dos fatores de risco identificados na história clínica do paciente, questionando a necessidade da realização do ECG pré-operatório.

Em nosso estudo, o sexo masculino e cirurgia de moderado/alto risco foram preditores independentes para desfechos adversos pós-operatórios na fase hospitalar. Uma significante maior prevalência de complicações em cirurgias de maior porte é facilmente compreensível, visto que os eventos adversos são devido a fatores inerentes ao paciente, mas também estão fortemente relacionados a complexidade dos procedimentos cirúrgicos. A explicação do gênero como fator de risco para eventos adversos é mais difícil. Acreditamos que pelo fato de os pacientes do sexo masculino serem mais velhos e haverem se submetido a intervenções de maior porte pode em parte explicar esses resultados. Ainda, podemos observar que não houve diferença de gênero entre o grupo A e o grupo B. Também não foi observada maior prevalência de anormalidades eletrocardiográficas no sexo masculino.

Nesse ensaio, nós também avaliamos a importância das alterações eletrocardiográficas no desfecho pós-operatório e não encontramos nenhuma diferença significante. Outros estudos também têm demonstrado esses mesmos resultados. ^6 , 21^

Até onde nós conhecemos, este é o primeiro estudo desenhado de forma prospectiva e randomizada a avaliar o papel do ECG pré-operatório sobre os desfechos adversos pós-operatórios durante a fase hospitalar em indivíduos acima de 50 anos submetidos à intervenção cirúrgica sob anestesia geral. Nossos resultados podem ter implicações práticas imediatas, ao ajudar algumas sociedades médicas a se reposicionarem sobre as recomendações de diretrizes em solicitar o ECG pré-operatório baseado apenas na idade dos pacientes.

As limitações desse estudo são: primeiro, consideramos o número de pacientes ainda pequeno frente o universo de procedimentos cirúrgicos realizados; segundo, esse estudo foi realizado em uma única instituição o que pode dificultar a extrapolação desses resultados para outras instituições com estrutura e profissionais distintos; terceiro, estudamos apenas uma população que possuía como diagnóstico de base uma doença neoplásica e sem alta prevalência de comorbidades, e em quarto, não foi feito o seguimento tardio desses pacientes após a alta hospitalar, escopo para outra análise.

## Conclusão

Os achados desse estudo sugerem que em pacientes com idade superior a 50 anos, sem comorbidades, submetidos à intervenção cirúrgica sob anestesia geral, o ECG pré-operatório não acrescenta benefício em predizer complicações pós-operatórias durante a fase hospitalar, sugerindo uma profunda reflexão sobre a real necessidade da solicitação de forma rotineira desse exame baseado apenas na faixa etária dos pacientes.
