# Supplementary material for: Importância Prognóstica do Eletrocardiograma Pré-Operatório em Pacientes de Baixo Risco Submetidos à Intervenção Cirúrgica sob Anestesia Geral
Source: Arq Bras Cardiol. 2024 Jan 24;121(1):e20230098. [Article in Portuguese] doi: 10.36660/abc.20230098 (PMC11098581; doi:10.36660/abc.20230098)
Supplement: Translation

# Translation: Prognostic Value of Preoperative Electrocardiogram in Low-Risk Patients Undergoing Surgical Intervention and General Anesthesia

**Authors:** Lafayete Ramos, Alexandre Chataubriand Coutinho, José Rebelato, Marcos Vinicius Ramos, Eliane Elly, Pedro Amoedo, Gustavo Viel, Valdir Ambrósio Moises

**Affiliations:** 1 Brazil Instituto Brasileiro de Controle do Câncer, São Paulo, SP – Brazil; 2 Brazil Universidade Federal de São Paulo, São Paulo, SP – Brazil; 3 Brazil Centro Universitário São Camilo, São Paulo, SP – Brazil; 4 Brazil Faculdade de Medicina de Marilia, Marília, SP – Brazil

**Keywords:** Electrocardiography, Preoperative Care, General Surgery

## Abstract

**Background:**

Patients aged over 50 years require four times more surgical interventions than younger groups. Many guidelines recommend the performance of preoperative electrocardiogram (ECG) in this population.

**Objectives:**

To determine the value of preoperative ECG in patients aged over 50 years and classified as ASA I–II (surgical risk).

**Methods:**

Patients older than 50 years, without comorbidities, who underwent surgical intervention and general anesthesia were included in the study. Patients were randomized to undergo ECG (group A, n=214) or not (group B, n=213) in the preoperative period. The following variables were analyzed: sex, age, ECG, chest x-ray and laboratory tests results, surgical risk, surgery duration, adverse events and in-hospital mortality. The level of significance was set at 5%.

**Results:**

Adverse outcomes were reported in 23 (5.4%) patients, with a significant number of adverse events in male patients (OR=7.91 95%CI 3.3-18.90, p<0.001) and in those undergoing major surgeries (OR=30.02 95%CI 4.01-224.92, p<0.001). No differences were observed between patients who underwent ECG and those who did not (OR=1.59, 95%CI, 0.67-3.75, p=0.289). No significant differences were found in the other variables. In multivariate logistic regression, male sex (OR = 6.49; 95%CI 2.42-17.42, p<0.001) and major surgery (OR=22.62; 95%CI 2.95-173.41, p=0.002) were independent predictors of adverse outcomes, whereas undergoing (or not) ECG (OR=1.09; IC95% 0.41-2.90, p=0.867) remained without statistical significance.

**Conclusion:**

Our findings suggest that preoperative ECG could not predict an increased risk of adverse outcomes in our study population during the hospital phase.

## Introduction

The number of non-cardiac surgeries has been increasing, surpassing 300 million interventions per year. ^1 , 2^ In developed countries, mortality rates have varied from 0.4 to 0.8% and complication rates from 3% to 16%, of which 40% seem to be related to the cardiovascular system. ^3 , 4^ People over the age of 50 require four times more surgical interventions than younger groups and, with the increasing aging of the population, especially due to greater attention paid to chronic diseases, it is estimated that these procedures exponentially increase in the next years. ^5^

Today, it is recommended a preoperative electrocardiogram (ECG) for people older than 40 years, since studies on surgical populations have shown that electrocardiographic changes increase with age, and several of them may have clinical implications in the anesthetic management. ^6 , 7^

In this context, in most institutions, clinical and laboratory evaluation has been routinely performed to determine patients’ preoperative status aiming at reducing morbidity and mortality. One of the tests used for clinical evaluation is ECG. This test is particularly ordered for patients aged older than 40, regardless of their clinical condition, at a variable level of recommendation (depending on the guideline used), but always with a weak level of evidence because of the scarcity of studies with sufficient design, quality, and sample size to allow a stronger recommendation. ^8 - 12^

The present study aims to assess, for the first time, using a prospective, randomized design, the need for a routine preoperative ECG in patients older than 50 years, without comorbidities, who had undergone non-cardiac surgery under general anesthesia.

## Methods

Between April 2020 and February 2022, 427 patients aged older than 50 years, without comorbidities, underwent non-cardiac surgery under general anesthesia. All patients had normal physical examination in the preoperative evaluation. Those who met the inclusion criteria and accepted to participate in the study after signing the consent form were randomized in a 1:1 proportion to undergo ECG or not.

Laboratory tests (complete blood count, glycemia, urea, creatinine and coagulogram) were performed in all patients, and all participants underwent posteroanterior chest X-ray and antineoplastic surgical procedure (under general anesthesia).

The following variables were analyzed – sex, age, ECG results (for those who underwent ECG), chest X-ray results, laboratory tests (complete blood count, glycemia, urea, creatinine and coagulogram). Data on preoperative surgical risk ( *American Society of Anesthesiology - ASA* ), duration of procedure, adverse events and in-hospital mortality were also analyzed. In-hospital mortality and morbidity rates of patients who underwent ECG (group A) were compared with those who underwent surgery without the ECG (group B). Group A patients were divided into two subgroups A1 (with normal ECG) and A2 (with abnormal ECG findings); these groups were compared with each other and with group B. The ECG was interpreted by senior cardiologist of the participating institution and was considered abnormal if there was abnormal (other than sinus) rhythm, presence of cardiac chamber overload, intraventricular or atrioventricular block, reversal of the T wave polarity in at least two contiguous leads, more than three atrial or ventricular premature beats, ventricular preexcitation or QTc>470ms. Chest x-ray was assessed by two radiologists at the radiology department; the presence of parenchyma infiltration, tuberculosis sequelae, pleural effusion, increased cardiac area or metastasis. Laboratory test values that were out of the normal range adopted by the central laboratory of the institution was considered abnormal. No patient was excluded from randomization due to any abnormal finding in the laboratory tests, chest x-ray or ECG.

Surgical procedures were divided into two groups, of low risk and of moderate/high risk. In this study, a high/moderate-risk surgery was defined as an intracavitary (cranial, chest, abdominal or pelvic) surgery and those procedures in which fluid replacement exceeded 30mL/Kg.

Adverse outcome was any type of complication (clinical and/or surgical) that prolonged the hospital length of stay predicted for each procedure, or death due to any cause. This was assessed both individually and combined (named as morbidity and mortality).

This study was approved by the ethics committee of the *Instituto Brasileiro de Controle de Cancer* (Brazilian Institute for Cancer Control) and by the ethics committees of participating institutions (CAAE 20728719.3.0000.0072)

### Statistical analysis

Qualitative characteristics were described as absolute and relative frequencies, by group (with or without ECG), and their associations were evaluated by chi-square test or by exact tests (Fisher’s exact test or the likelihood-ratio test). Quantitative features were described as summary measures (mean, standard deviation, median and interquartile range) and compared by unpaired Student’s t-test or Mann-Whitney test, according to normality of distribution assessed by Kolmogorov-Smirnov test.

Morbidity and mortality were described according to each quantitative and qualitative feature using the same tests above mentioned.

The variables that showed statistical significance by Fisher exact test or the likelihood-ratio test, or by the Student’s t-test/Mann-Whitney test regarding morbidity and mortality were included in the multivariate analysis by logistic regression. ECG was also included in the analysis to evaluate whether the test affected morbimortality.

The software SPSS for Windows, version 22.0, was used for statistical analysis, and the significance level was set at 5%.

## Results

This study included 427 patients (83.6% women) aged over 50 years, with solid tumors and no history of comorbidities, who underwent elective surgeries under general anesthesia. The ECG group patients (group A) were older, had more abnormal laboratory findings and longer operative time during surgery. The other variables did not show statistical significance ( Table 1 ).

**Table 1:** – Analysis of patients undergoing surgery with or without preoperative electrocardiogram (ECG)

Variable	ECG						

	Without		With		Total		p
	
	n	%	n	%	n	%	
**Sex**							0,199
Male	30	14.1	40	18.7	70	16.4	
Female	183	85.9	174	81.3	357	83.6	
**Age (years)**	57.37±6.16		58.71±7.51		427	100.0	0.044 ^#^
Surgical risk (major/minor surgery)							0.225
Low risk	123	57.7	110	51.9	233	54.8	
Moderate/high risk	90	42.3	102	48.1	192	45.2	
**Chest x-ray**							0.192
Normal	208	97.7	204	95.3	412	96.5	
Abnormal	5	2.3	10	4.7	15	3.5	
**Laboratory**							<0.001
Normal	213	100.0	199	93.0	412	96.5	
Abnormal	0	0.0	15	7.0	15	3.5	
**Surgical risk (ASA)**							0.372*
I	212	99.5	210	98.1	422	98.8	
II	1	0.5	4	1.9	5	1.2	
**Time of surgery (hours)**	2 (2 – 3)		2 (2 – 4)		427	100.0	0.042 ^$^
**Mortality**							>0.999*
No	212	99.5	212	99.1	424	99.3	
Yes	1	0.5	2	0.9	3	0.7	
**Complications resolved**							0.193
No	205	96.2	200	93.5	405	94.8	
Yes	8	3.8	14	6.5	22	5.2	
**Morbimortality**							0.289
No	204	95.8	200	93.5	404	94.6	
Yes	9	4.2	14	6.5	23	5.4	
**Total**	213	100	214	100	427	100	

Chi-square test; * Fisher’s exact test; # Student’s t-test; $ Mann-Whitney test (interquartile range)

Adverse outcomes occurred in 23(5.4%) patients, including three deaths (0.7%). There was a significant number of adverse events in male patients and in those who underwent moderate/high-risk interventions, with no difference in postoperative complications between patients with ECG and without ECG ( Central Illustration ). No statistically significant differences were found in age, abnormal chest x-ray or laboratory findings, surgical risk (ASA) and surgery duration ( Table 2 ).

**Central Illustration:** : Prognostic Value of Preoperative Electrocardiogram in Low-Risk Patients Undergoing Surgical Intervention and General Anesthesia

**Table 2:** – Analysis of postoperative outcomes in relation to the study variables

	Morbimortality							
	
Variable	No		Yes		OR	95%CI		p
		
	n	%	n	%		Inferior	Superior	
**Sex**								<0.001*
Male	57	81.4	13	18.6	7.91	3.31	18.90	
Female	347	97.2	10	2.8	1.00			
**Age (years)**	56 (52 – 62)		57 (55 – 60)		1.01	0.95	1.07	0.754
**Surgical risk (major/minor surgery)**								<0.001
Low risk	232	99.6	1	0.4	1.00			
Moderate/High risk	170	88.5	22	11.5	30.02	4.01	224.92	
**Electrocardiogram**								0.289
No	204	95.8	9	4.2	1.00			
Yes	200	93.5	14	6.5	1.59	0.67	3.75	
**Chest x-ray**								>0.999*
Normal	389	94.4	23	5.6	1.00			
Abnormal	15	100.0	0	0.0	&			
**Laboratory**								0.190*
Normal	391	94.9	21	5.1	1.00			
Abnormal	13	86.7	2	13.3	2.86	0.61	13.52	
**Surgical risk**								>0.999*
I	399	94.5	23	5.5	1.00			
II	5	100.0	0	0.0	&			
**Time of surgery (hours)**	2 (2 – 3)		2 (2 – 5)		1.30	1.01	1.67	0.193 ^$^
**Total**	404	94.6	23	5.4				

Chi-square test; * Fisher’s exact test; # Student’s t-test; $ Mann-Whitney test (interquartile range); CI: confidence interval; & estimation was not possible

Multivariate logistic regression analysis showed that male sex and surgical risk (minor/major surgery) were predictors of adverse outcomes in the postoperative period in this population, whereas ECG did not show statistical significance ( Table 3 ).

**Table 3:** – Predictors of morbidity and mortality in the study population: multivariate logistic regression analysis

Variable	OR	95%CI		p

		Inferior	Superior	
Sex (male)	6.49	2.42	17.42	<0.001
Age (years)	1.00	0.93	1.07	0.943
Major surgery (Moderate/high risk)	22.62	2.95	173.41	0.003
Abnormal laboratory findings	1.39	0.22	8.88	0.726
Time of surgery	1.47	1.09	1.99	0.013
Electrocardiogram performed	1.09	0.41	2.90	0.867

CI: confidence interval

We also evaluated whether the presence of abnormal electrocardiographic findings would have an impact on postoperative morbimortality. In the comparison of patients with abnormal ECG findings with those with normal ECG and those that did not undergo ECG (group B), no differences were found regarding the occurrence of adverse events ( Table 4 ). In addition, no difference was observed between patients with abnormal ECG findings and those with normal ECG in group A ( Table 5 ).

**Table 4:** – Postoperative outcomes in patients with normal electrocardiogram (ECG) and abnormal ECG, as compared with patients without ECG

Variable	ECG								

	Normal		Abnormal		Without ECG		Total		p

	n	%	n	%	n	%	N	%	
**Mortality**									0.241
No	196	99.5	16	94.1	212	99.5	424	99.3	
Yes	1	0.5	1	5.9	1	0.5	3	0.7	
**Morbidity**									0.300
No	185	93.9	15	88.2	205	96.2	405	94.8	
Yes	12	6.1	2	11.8	8	3.8	22	5.2	
**Morbimortality**									0.402
No	185	93.9	15	88.2	204	95.8	404	94.6	
Yes	12	6.1	2	11.8	9	4.2	23	5.4	
Total	197	100	17	100	213	100	427	100	

Likelihood-ratio test

**Table 5:** – Comparison of postoperative adverse outcomes between patients with normal electrocardiogram (ECG) and patients with abnormal ECG

Variable	ECG				

	Normal		Abnormal		p
	
	n	%	n	%	
**Mortality**					0.153
No	196	99.5	16	94.1	
Yes	1	0.5	1	5.9	
**Morbidity**					0.307
No	185	93.9	15	88.2	
Yes	12	6.1	2	11.8	
**Morbimortality**					0.307
No	185	93.9	15	88.2	
Yes	12	6.1	2	11.8	
Total	197	100.0	17	100.0	

*Likelihood-ratio test*

Male patients were older and submitted to higher risk surgery. With respect to the other variables studied, no differences were observed between men and women ( Table 6 ).

**Table 6:** – Characteristics of patients by sex

Variables	Sex				

	Male		Female		p
	
	n	%	n	%	
**ECG**					0.104 ^#^
Normal	34	48.6	163	45.7	
Abnormal	6	8.6	11	3.1	
Without ECG	30	42.9	183	51.3	
**Surgical risk (minor/major surgery)**					<0.001
Low risk	24	34.8	209	58.7	
Moderate/high risk	45	65.2	147	41.3	
**Time of surgery (h)**	2 (1 – 3)		3 (2 – 3)		0.067 ^&^
Chest x-ray					
Normal	69	98.6	343	96.1	0.483*
Abnormal	1	1.4	14	3.9	
**Laboratory**					0.081*
Normal	65	92.9	347	97.2	
Abnormal	5	7.1	10	2.8	
**Surgical risk (ASA)**					0.593*
I	69	98.6	353	98.9	
II	1	1.4	4	1.1	
**Age (years)**	60.9±8.7		57.5±6.3		0.002 ^$^
**Total**	70	100.0	357	100.0	

Chi-square test; * Fisher’s exact test; # Likelihood-ratio test; $Student’s t-test; & Mann-Whitney test (interquartile range); ASA: American Society of Anesthesiology

## Discussion

Prognostic meaning of preoperative ECG has changed in the last decades. Since the end of the 70s, resting ECG has been widely used as a marker of cardiovascular risk in individuals undergoing elective surgery. Electrocardiographic changes, such as the presence of pathological Q waves and arrythmias are included in the Goldman risk score. ^13^ These results were repeated, confirming the prognostic value of ECG in the preoperative period. ^14 , 15^

Payne et al. ^16^ evaluated 345 patients of a prospective cohort and concluded that ECG is a useful test to predict perioperative cardiovascular events. Other studies ^17 - 19^ showed that abnormal ECG in the preoperative period could predict cardiovascular complications especially in the presence of prolonged QT interval. However, these favorable results were later questioned by other authors, ^6 , 20 , 21^ and such controversy still remains today. Many of these questions could have been answered if there were robust evidence from prospective, randomized trials with patients undergoing elective surgery and general anesthesia.

The main finding of our study was that patients without comorbidities, even those aged over 50 years (mean age of 58 years) undergoing surgical intervention and general anesthesia may not benefit from preoperative ECG. We did not observe differences in the percentage of adverse events between group A (with ECG) and group B (without ECG). There was a trend for higher morbidity and mortality in group A, which may be explained by the fact that these patients were older, had more abnormal laboratory findings and having been undergone longer surgeries.

Some studies corroborate our findings. Richardson et al., ^21^ evaluating retrospectively a cohort of 152,479 patients, concluded that preoperative ECG is not valuable to predict postoperative infarction or cardiovascular mortality. Similarly, Liu et al., ^6^ in a study with 513 patients, observed that electrocardiographic abnormalities could not predict cardiovascular complications in the elderly population and hence ECG was not useful in these patients. In addition, van Klein et al., ^20^ in an observational study, evaluated the use of ECG in 2967 patients older than 50 years and found that although the presence of intraventricular block is correlated with the risk of postoperative infarction and death, it did not improve prediction beyond risk factors identified on patient history, thereby questioning the need for a preoperative ECG.

In our study, male sex and moderate/high-risk surgery were independent predictors of adverse postoperative outcomes during hospitalization. A significantly higher prevalence of complications in major surgeries is easily understandable, since adverse events not only result from factors inherent to the patient, but also are strongly correlated with the complexity of surgeries. The explanation of the role of male sex as a risk factor for adverse events, however, is more difficult. We believe that the fact that male patients were older and that they had undergone more extensive surgeries may, in part, explain these results. Also, there was no difference in sex distribution between group A and group B. We also did not observe higher prevalence of electrocardiographic abnormalities among men than women.

In this study, we also evaluated the role of electrocardiographic changes in the postoperative outcome and did not find any statistically significant difference. This is in accordance with results of previous studies. ^6 , 21^

To our knowledge, this is the first prospective and randomized study to evaluate the role of preoperative ECG in adverse postoperative outcomes in the hospital phase in individuals older than 50 years who underwent surgical intervention under general anesthesia. Our results may have immediate practical implications by helping some medical societies in positioning themselves about guidelines recommendations for indicating preoperative ECG based on patients’ age only.

Limitations of the study include the relatively small number of patients in face of the multitude of surgical interventions; second, this was a single-center study, which may make extrapolation of results to other institutions with different infrastructure and staff difficult; third, we studied a single population with underlying diagnosis of cancer and without a high prevalence of comorbidities; and finally, a long-term follow-up of these patients after discharge was not performed, which may be the scope of future research.

## Conclusion

The findings of the present study suggest that in patients older than 50 years, without comorbidities, undergoing surgical intervention and general anesthesia, preoperative ECG does not add value in predicting postoperative complications in the hospital phase, suggesting the necessity for a deep analysis on the real need for ordering this test in a routine basis, considering patients’ age only.
